# Supramolecular Interactions of Terpyridine-Derived Cores of Metallomesogen Precursors

**DOI:** 10.3390/ijms141020729

**Published:** 2013-10-15

**Authors:** Young Hoon Lee, Jack M. Harrowfield, Jong Won Shin, Mi Seon Won, Elisabeth Rukmini, Shinya Hayami, Kil Sik Min, Yang Kim

**Affiliations:** 1 Department of Chemistry and Advanced Materials, Kosin University, 149-1, Dongsam-dong, Yeongdo-gu, Busan 606-701, Korea; E-Mails: dasis75@hanmail.net (Y.H.L.); wonmiseon@hanmail.net (M.S.W.); 2 Institut de Science et de l’Ingénierie Supramoléculaires, Université de Strasbourg, 8, allée Gaspard Monge 67083, Strasbourg, France; 3 Department of Chemistry, Graduate School, Kyungpook National University, Daegu 702-701, Korea; E-Mails: love2wony@hanmail.net (J.W.S.); minks@knu.ac.kr (K.S.M.); 4 School of Medicine, Atma Jaya Catholic University of Indonesia, Pluit Raya #2, Jakarta 14440, Indonesia; E-Mail: elisabeth.rukmini@atmajaya.ac.id; 5 Department of Chemistry, Graduate School of Science and Technology, Kumamoto University, 2-39-1 Kurokami, Kumamoto 860-8555, Japan; E-Mail: hayami@sci.kumamoto-u.ac.jp

**Keywords:** Hirshfeld surfaces, crystal structures, labile interactions, functionalised terpyridines, spin crossover

## Abstract

Use of Hirshfeld surfaces calculated from crystal structure determinations on various transition metal ion complexes of three terpyridine ligands carrying trimethoxyphenyl substituents has enabled an assessment of the contribution made by the ligand components to the interactions determining the lattice structures, interactions expected also to be present in metallomesogens derived from similar ligands. The form of the link joining the trimethoxyphenyl substituent to the 4′ position of 2,2′;6′,2″-terpyridine is of some importance. In the case of the Co(II) complexes of two of the ligands, their spin-crossover characteristics can be rationalised in terms of the different interactions seen in their lattices.

## 1. Introduction

**Figure 1:**
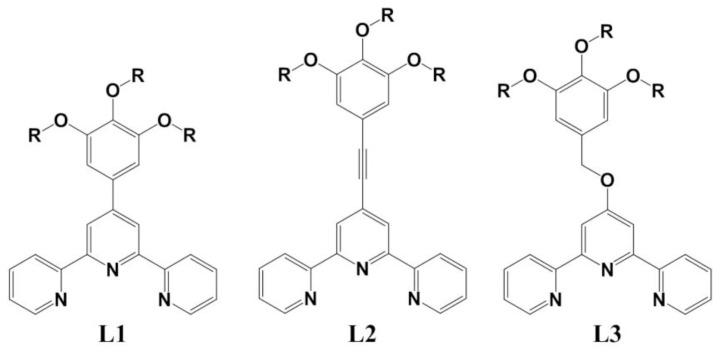
Ligands used to form the crystalline complexes described in the present work, for which R = CH_3_.

The effectiveness of 2, 2,2′;6′,2″-terpyridine as a ligand and the variety of functionality which may be introduced through substitution on this core [1] have engendered intense interest in the coordination chemistry and possible applications of this ligand and its derivatives [2]. One aspect of this chemistry which has been a particular concern of the present authors and their collaborators is the induction of mesomorphic behaviour in Co(II) and Fe(II) complexes as a means of controlling their magnetic properties involving “spin crossover” [3,4]. Very recently, it has been shown that the Co(II) complex of ligand **L1** (Figure 1; R = C_16_H_33_), for example, undergoes a high-spin to low-spin change in magnetism which is triggered by a crystal- to-mesophase transition [5]. In this case, as in many others, [6] the introduction of a gallate- like substituent with three hexadecyloxy groups at the 4′ position of terpyridine proved sufficient to induce mesomorphism, here in the Co(II) complex as its tetrafluoroborate salt, while the analogous complex with short methoxy substituents showed only a single crystalline phase. Nonetheless, since liquid crystal behaviour in general is determined not only by interactions involving flexible alkyl-chain units but also by those involving more rigid units such as aromatic rings [7], the crystal structure [5] of this non-mesomorphic complex was of interest for its use in defining modes of interaction between core groups of the complex which could presumably influence the behaviour of the mesomorphic species. An interesting feature of the lattice of the complex was the loss of the “terpyridine embrace” [8,9] ascribed to aromatic–aromatic interactions [8–10] and which is a striking feature of the lattice of most M(II) complexes of unsubstituted terpyridine, although its loss may well be common to most such complexes of terpyridines with large substituents [11]. To explore further the role of a 3,4,5-trimethoxyphenyl substituent in determining the labile interactions of metal complexes of 4′-substituted terpyridine, we have structurally characterised not only other metal ion complexes of **L1** but also various metal ion complexes of the related ligands **L2** and **L3** (Figure 1) and report the essential aspects of this work herein. The nature of the link between the terpyridine and trimethoxyphenyl units has proven to be of some significance.

## 2. Results and Discussion

**Figure 2:**
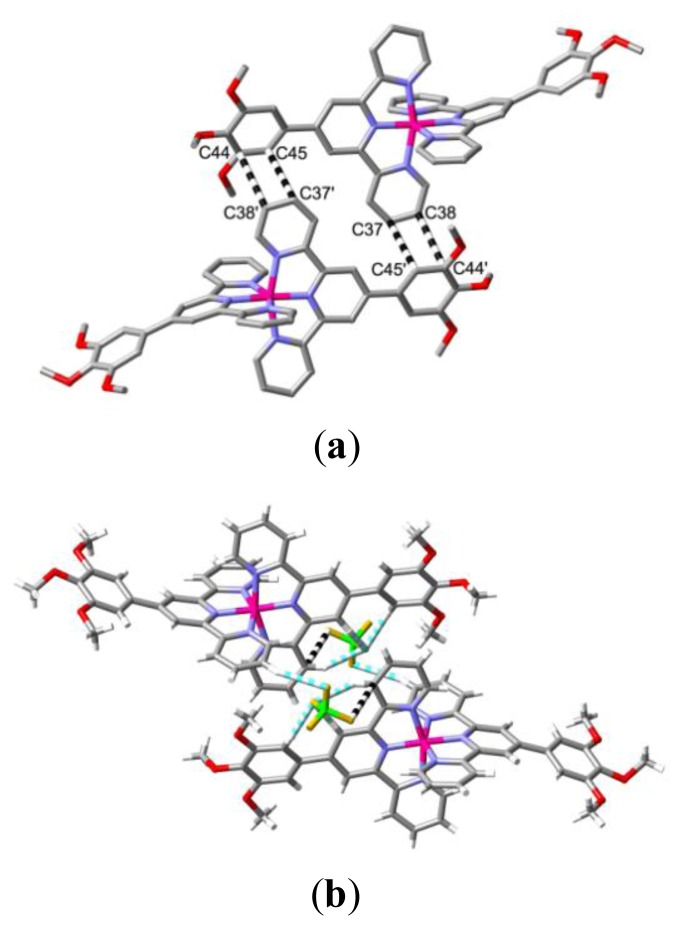
(**a**) Reciprocal π-type interactions (dashed lines) between trimethoxyphenyl- and pyridyl-group carbon atoms of cations (stick representations; H-atoms not shown) with Co…Co 10.621(3) Å within the lattice of [Co(**L1**)_2_](BF_4_)_2_·CH_3_CN·0.5H_2_O; (**b**) Bridging of the same pair of cations as in (a) via interactions with tetrafluoroborate anions of both CH…F (blue and white dashed lines) and F…π (black and white dashed lines) types.

**Figure 3:**
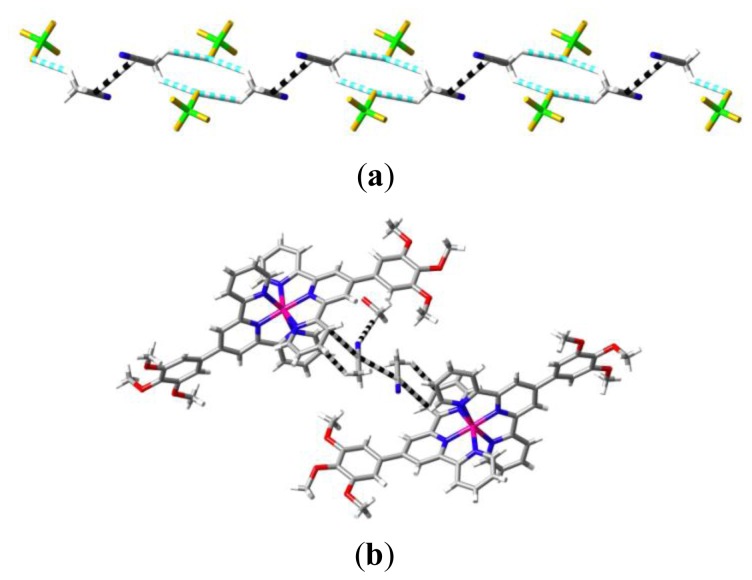
(**a**) Portion of the chain formed by bridging of pairs of antiparallel CH_3_CN dipoles by CH…F interactions (blue and white dashed lines) with F…H 2.570(2) and 2.564(2) Å for the two bonds of a given F. The nitrile-C…C-nitrile contacts, 3.267(6) Å, are shown as black and white dashed lines; (**b**) π-interactions involved in the bridging of two cations with Co…Co 13.947(6) Å by every one of the CH_3_CN pairs shown in (**a**). One of the nitrile-*N*…HC interactions involving a methoxyl group of a third adjacent cation is also shown.

Several features of the structure of [Co(**L1**)_2_](BF_4_)_2_·CH_3_CN·0.5H_2_O [5] provide useful reference points for analysis of all the structures presently reported. It might, of course, be anticipated that the presence of electron-rich trialkoxyphenyl and electron-poor coordinated pyridyl units would lead to significant donor–acceptor interactions between them in the solid state but there is minimal evidence of such in this particular case. Thus, for two cations where the separation Co…Co is 10.621(3) Å, there are two contacts between trimethoxyphenyl and terpyridine units (C37…C45′ 3.376(3); C38…C44′ 3.386(3) Å, symmetry operation ‘ x-1, 1-y, 1-z; Figure 2(a)) which can be considered indicative of π–π/donor–acceptor interactions but the rings involved are far from parallel and do not overlap in projection. As well, the two cations can be considered to be bridged by interactions with tetrafluoroborate anions (involving B2 only) of both CH…F and F…π types (Figure 2(b)). For pairs of cations with a Co…Co separation of 12.021(3) Å, there are slightly longer reciprocal contacts C(7)…C(16′) of 3.428(4) Å, but here the Hirshfeld surfaces obtained with CrystalExplorer [12] do not indicate that significant interactions occur and indeed the ligand units involved are bowed in such a way as to minimise contacts and once again the cations are bridged by multiple interactions with the anions. Cation–anion attraction is expected to be the dominant force in lattices of complexes of the present type [8,9] but as the charges are delocalised, it is convenient to treat its effects as charge enhancement of various pairwise interactions which can be identified by using, as here for example, CrystalExplorer [12]. There is particularly strong current interest in anion interactions with electron deficient aromatic systems in general [13–16]. In [Co(**L1**)_2_](BF_4_)_2_·CH_3_CN·0.5H_2_O, the primary role of cation–anion interactions is indicated by the fact that the lattice contains chains of cations separated by *a* (9.5623(3) Å), the shortest Co…Co separation in the lattice, with the cations linked as a result of interactions (CH…F and F…π but see also ahead) with both (inequivalent) anions and certainly not as a result of terpyridine embraces, unlike unsubstituted terpyridine systems where the shortest cation separations are almost always associated with such embraces [8,9,11]. Interestingly, the inequivalent anions can be simply distinguished through their interactions with lattice solvent, the anion involving B1 being associated with acetonitrile while that involving B2 is associated with water. Disorder and partial occupancy of the water molecule sites renders description of the latter situation complicated but the former involves chains in which pairs of acetonitrile molecules in an antiparallel dipolar arrangement are bridged by CH…F interactions with pairs of anions (Figure 3a). Separate anions in these chains form F…H bonds with the cations separated by 9.5623(3) Å, thus creating indirect anion bridging in addition to direct links to one anion. Analysis of the interactions of the acetonitrile molecules using CrystalExplorer indicates that the principal interaction between the two in a pair involves the nitrile-C atoms and that these are also involved in π-type interactions with a pyridine ring carbon atom each from cations 13.947(6) Å (Co…Co) apart, while the methyl groups have a strong CH…π interaction with the adjacent pyridine-ring carbon atoms (Figure 3b). The nitrile-N atom appears to be involved in a weak N…HC bond involving a methoxy group of a cation which is not one of the pair linked by the nitrile-C interactions. Clearly, there are many forces competing with any direct interaction of phenyl and pyridyl rings!

**Figure 4:**
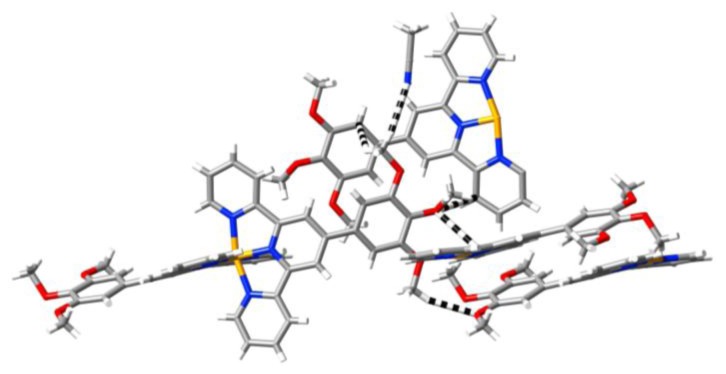
Partial representation of the interactions of methoxy groups on one terminus of a cation within the lattice of [Fe(**L1**)_2_](ClO_4_)_2_·CH_3_CN. For clarity, only half each of the three other cations containing groups in interaction with those of the first are shown. The contacts indicated by the black and white dashed lines involve CH…O, CH…N, CH…π and O…π interactions.

Given that the metal ions are encased in a large ligand sheath and that perchlorate and tetrafluoroborate are tetrahedral anions of similar size, it is unsurprising that [Fe(**L1**)_2_](ClO_4_)_2_·CH_3_CN and [Ni(**L1**)_2_](ClO_4_)_2_·CH_3_CN are essentially isostructural with [Co(**L1**)_2_](BF_4_)_2_·CH_3_CN·0.5H_2_O. Thus, there is again minimal evidence for a significant role of aromatic–aromatic, specifically phenyl–pyridyl, interactions in defining the lattice of these complexes, although the formation of bridges via π-type interactions between acetonitrile pairs and pyridyl rings is a feature of all three structures (with perchlorate H–bond bridging of the acetonitrile pairs being slightly further from symmetrical than that of tetrafluoroborate). Examination of the three structures using CrystalExplorer shows that the role of the trimethoxyphenyl groups in lattice construction is largely associated with the methoxy groups, principally through O…π interactions and H-bond acceptance by the oxygen atoms and H-bond donation, including CH…π, by the methyl groups, some examples of these interactions being shown for [Fe(**L1**)_2_](ClO_4_)_2_·CH_3_CN in Figure 4.

**Figure 5:**
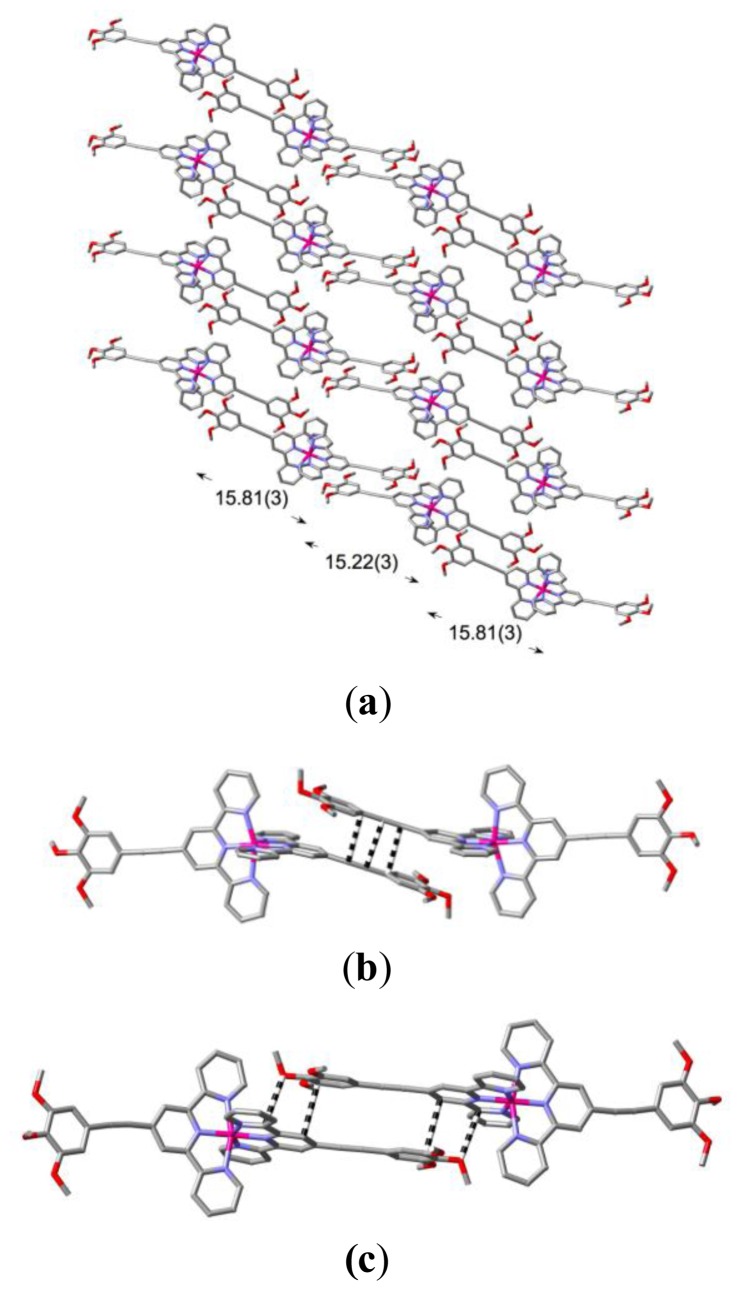
(**a**) A partial view of the sheet of cations lying parallel to the (0 1 2) plane in the lattice of [Co(**L2**)_2_](ClO_4_)_2_·H_2_O, showing the side-by-side chains of cations apparently linked through stacking interactions; (**b**) Contacts within the closer, centrosymmetric pairs of cations of a given chain indicated from the Hirshfeld surface to be significant π interactions. Alkynyl-C…C-alkynyl is 3.296(7), alkynyl-C…C-aromatic is 3.262(7) Å; (**c**) Weaker π-type interactions within the more remote centrosymmetric pair of a given chain—O…C 3.141(6), C…C 3.357(7) Å.

Insertion of a triple-bond link between the terpyridine and trimethoxyphenyl units as in **L2** produces a lattice of [Co(**L2**)_2_](BF_4_)_2_·H_2_O which is very similar to those just described for **L1** complexes. The space group is again *P-1* and the shortest Co…Co separation of 9.177(3) Å, significantly shorter than that of [Co(**L1**)_2_](BF_4_)_2_·CH_3_CN·0.5H_2_O, is again found in pairs (isolated in this case) linked by aromatic-CH…FBF…HC-aromatic bridges, here coupled with aromatic-CH…O(water)…FBF…HC-aromatic bridges, with no “terpyridine embrace” evident. Viewed down *a*, the lattice has a very similar appearance to that of [Co(**L1**)_2_](BF_4_)_2_·CH_3_CN·0.5H_2_O in that sheets can be seen edge-on, sheets in which cation chains lie side by side, very much as seen in various complexes of terpyridine with simple polyphenyl substituents [11,17]. In [Co(**L2**)_2_](BF_4_)_2_·H_2_O, these sheets lie parallel to the (0 1 2) plane and the chains (running parallel to the *ac* diagonal) within these sheets (Figure 5a) give the impression of being linked through stacking arrays of the substituted terpyridine units. In fact, the Co…Co separations in these chains alternate between 15.22(3) and 15.81(3) Å and it is only for the closer pairs that the use of CrystalExplorer (Hirshfeld surfaces) provides evidence of significant interactions involving the triple bond carbon atoms and the carbon of the trimethoxyphenyl group to which they are linked (Figure 5b). These pairs also happen to be bridged by CH…F interactions with the tetrafluoroborate anions and while the pairs showing no triple-bond carbon stacking interactions also interact with the anions, this does not involve any bridging. There are, nonetheless, some apparently weaker, bridging π-stacking interactions involving O…C and C…C contacts (Figure 5c). An obvious interpretation of these observations is that the stronger cation–anion interactions can sometimes enforce π–π contacts of significance. In the present instance, the alkynyl-C contacts may be of significance in explaining the anomalous magnetic properties of [Co(**L2**)_2_](BF_4_)_2_·H_2_O, where heating and cooling cycles produce continuous changes (Figure S1) which might be due to cyclisation of the CC units in contact, though this remains to be investigated further. Note that the first heating cycle for this complex gives a susceptibility curve consistent with a “gradual” spin crossover, similar to that seen for [Co(**L1**)_2_](BF_4_)_2_·CH_3_CN·0.5H_2_O [5] and as expected [9] given the relatively long Co…Co separations and the apparent lack of π-contacts of terpyridine units.

**Figure 6:**
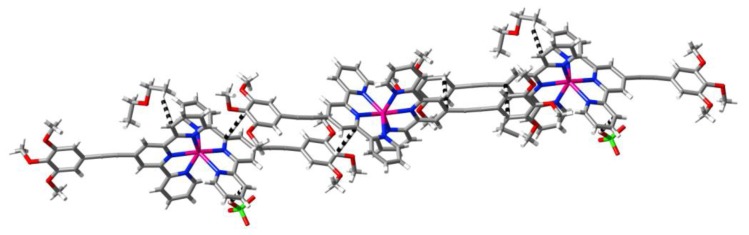
Partial view of a chain of cations within the lattice of [Cd(**L2**)_2_](ClO_4_)_2_·CH_3_CN·Et_2_O showing, as black and white dashed lines, π interactions which involve the lattice solvent and anions as well as aromatic rings. All C…C contacts (3.31(7) Å) are the same within experimental error, while H_2_CH…C-aromatic is 2.78(4) Å and O_3_ClO…C-aromatic is 3.08(6) Å.

While the complex [Cd(**L2**)_2_](ClO_4_)_2_·CH_3_CN·Et_2_O has a somewhat different composition to its Co(II) analog, it also crystallises in space group P-1 and the lattice is very similar to that just described, except that the cation sheets lie parallel to the (0 1 −1) plane. The shortest Cd…Cd separation is 9.73(2) Å, but there are other pairs with a separation of 9.90(2) Å and in fact all the cation pairs here can be considered part of a chain with alternating links of the slightly different distances resulting from bridging by differently oriented perchlorate anions. In the sheets parallel to (0 1 −1), it is again possible to discern chains of cations with overlapping aromatic units, here with Cd…Cd alternating between 15.51(3) and 15.56(3) Å, but what is striking is that there is now no indication of stacking interactions involving the triple-bond carbon atoms (their separations being ~0.15 Å greater than in the Co(II) complex). There are various contacts indicative of π-type interactions but only some of these involve the facing aromatics (Figure 6) and once again it can be argued that aromatic–aromatic interactions adjust readily to other forces controlling the lattice, so that seemingly dramatic changes may be associated with minor dimensional changes in the overall structure.

**Figure 7:**
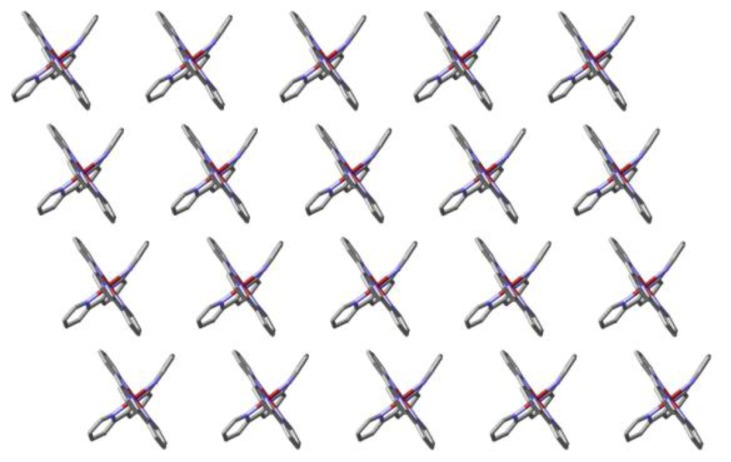
A partial view of a sheet of cations in the lattice of [Cu(**L3**)_2_](ClO_4_)_2_·CH_3_CN lying parallel to the ac plane, showing the apparent partial retention of a terpyridine embrace in the sense that terpyridine units lie in relatively close parallel planes. To emphasize this arrangement, the trimethoxyphenylmethyleneoxy substituents (as well as lattice solvent and anions) are not shown.

The lattice of [Cu(**L3**)_2_](ClO_4_)_2_·CH_3_CN, once again belonging to space group *P-1*, can be considered as composed of slightly undulating sheets of cations lying parallel to the *bc* plane and separated by layers containing the acetonitrile molecules and perchlorate anions. (Figure S2) Viewed down *b*, the acetonitrile molecules appear to form pairs with antiparallel dipoles having perchlorate neighbours but in fact the array is quite different to that seen in [Fe(**L1**)_2_](ClO_4_)_2_·CH_3_CN and [Ni(**L1**)_2_](ClO_4_)_2_·CH_3_CN, with no indication of any of the π-type interactions seen in those cases (and in the lattice of [Co(**L1**)_2_](BF_4_)_2_·CH_3_CN·0.5H_2_O), confirming thus their secondary importance. A possibly significant feature of the hydroxymethylene link between the aromatic units of **L3** is that it appears to allow not only bending of the mean plane of the ligand, as seen for one ligand of the two on a metal ion in all the present cases (an asymmetry noted in various related systems [3,4,11]) but also a more marked twisting of one trimethoxyphenyl group relative to the terpyridine unit to which it is attached (Figure S3. One feature of the lattice of [Cu(**L3**)_2_](ClO_4_)_2_·CH_3_CN that this additional flexibility may explain is the retention of a vestigial “embrace” array (Figure 7), although at most this involves reciprocal CH…π contacts (C2…H27′ 2.82(4) Å) and none of C…C (“OFF” [7]) type between terpyridine units.

**Figure 8:**
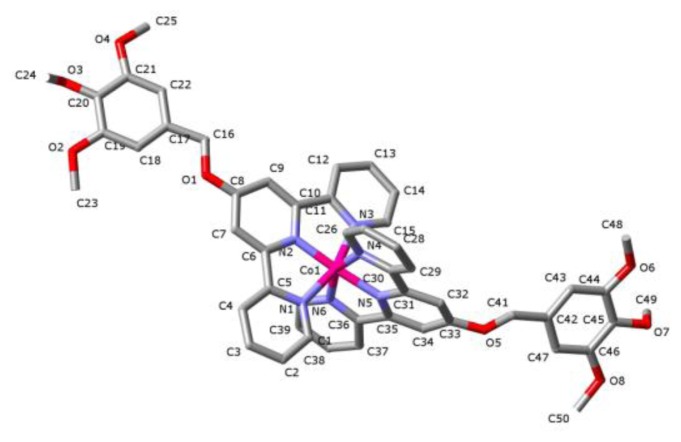
A view (stick representation; H-atoms omitted) of the cation present in the lattice of [Co(**L3**)_2_](BF_4_)_2_ at 100(2) K, with atom labelling shown as used in the text.

The crystal structure of [Co(**L3**)_2_](BF_4_)_2_ was determined at two temperatures (100(2) and 294(2) K) corresponding, on the basis of magnetic susceptibility measurements (see ahead), to low- and high-spin species, respectively. The same space group, *P-1*, was retained at both temperatures, so the spin state change is not complicated by phase changes. The change in size of the Co(II) centre due to the change in spin state is accommodated in bond length changes (Table 1) largely associated with the donor atoms of but one of the two ligands, another reason for the unsymmetrical form of the cation found in the solid state (Figure 8), although this lack of symmetry is found in both species and indeed in all the complexes presently described, so that it must also have more general origins (for example, the mismatch in cation and anion binding site geometry for labile interactions). Although the lattices of the Co(II) complex differ significantly from that of the Cu(II) species, it is again possible to discern partial arrays resembling that of the terpyridine embrace. For the low-spin Co(II) complex, application of the Co..Co ≤ 10 criterion for magnetic interactions [11] leads to the identification of pairs of cations where Co..Co is 9.37(1) Å and in these pairs there are close-to-parallel flanking pyridyl rings of the terpyridine units for which the use of CrystalExplorer indicates that there are reciprocal CH…π interactions (C13…H28′ 2.94(4) Å; symmetry operation ‘ = 1−x,1−y,z−1). The only C…C π-type interaction apparent on the Hirshfeld surface is that between C2 and C43′ (3.21(5) Å apart) in the pairs of cations where Co…Co is 10.23(3) Å. Note that this is an interaction between trimethoxyphenyl and pyridyl ring carbon atoms, not one between carbon atoms which could both be regarded as partial spin carriers [11]. There is also a strong π-type interaction of C10 and C11 with O3′ in a pair of cations where Co…Co is 15.64(4) Å, again one which does not bring spin centres into close proximity. As might be expected, an increase in temperature by nearly 200 K results in some fairly substantial rearrangement of the lattice but in high-spin [Co(**L3**)_2_](BF_4_)_2_ all major cation–anion interactions via aromatic-CH…F bonding are retained and it is essentially all π-type interactions (including O…π) which are lost or weakened. Thus, the only C…C interaction apparent is one still involving C43 but now with C14′ (C43…C14′ 3.32(6) Å) for cations where Co…Co is 10.22(3) Å. The rings involved are not parallel.

**Figure 9:**
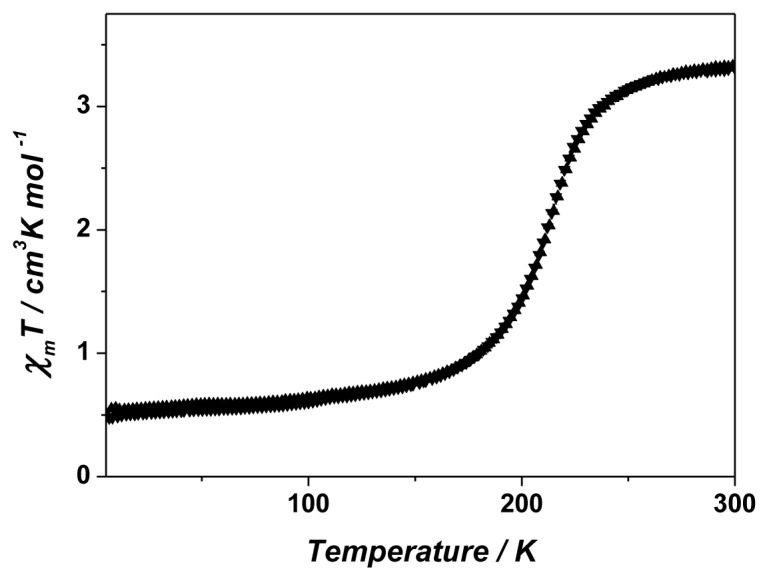
Temperature-dependent magnetic susceptibility measurements for [Co(**L3**)_2_](BF_4_)_2_.

Somewhat unexpectedly, the magnetic properties of [Co(**L3**)_2_](BF_4_)_2_ proved to be rather different to those of [Co(**L1**)_2_](BF_4_)_2_·CH_3_CN·0.5H_2_O, with a spin state change occurring quite abruptly, though without any hysteresis (Figure 9). This greater cooperativity for spin state change in [Co(**L3**)_2_](BF_4_)_2_ may, as we have suggested for various related systems, [11] reflect the ease of distortion of the lattice, here due to the enhanced flexibility of **L3** compared to **L1** noted above and also the lack of the rather unusual specific interactions involving lattice acetonitrile seen in [Co(**L1**)_2_](BF_4_)_2_·CH_3_CN·0.5H_2_O. The fact that there are numerous points of interaction possible for cation and anion and thus that stabilisation can be achieved in multiple arrangements may be a general factor influencing spin state changes in all the Co(II) complexes presently considered.

## 3. Experimental

### 3.1. Materials, Software and Instrumentation

Reagents were purchased from Wako and Aldrich and used without further purification. Structural data for the complexes were analysed using CrystalExplorer [12] and figures illustrating atom contacts prepared using CrystalMaker [18]. Microanalysis was performed at the Instrumental Analysis Centre of Kumamoto University. ^1^H NMR spectra were recorded on either a Bruker AM300 or a JEOL 500-ECX spectrometer at ambient temperature using deuterated solvents with TMS as internal reference. Magnetic susceptibilities of ground samples were measured on a superconducting quantum interference device (SQUID) magnetometer (Quantum Design MPMS-XL) under an applied field of 10^4^ Oe. Samples were placed in a gelatin capsule, mounted inside a straw and then fixed to the end of the sample transport rod.

### 3.2. Synthesis

Ligand **L1** was prepared as described earlier [4]. [2,2′:6′,2″]terpyridine-4′-ol [19], 2,6-di(pyridin-2-yl)pyridin-4-yl trifluoromethane-sulfonate [20], 5-ethynyl-1,2,3-trimethoxybenzene [21], and 5-(bromomethyl)-1,2,3-trimethoxybenzene [22] were prepared by literature procedures.

Ligand **L2**: A three-necked, round-bottomed flask was charged with 2,6-di(pyridin-2-yl)pyridin-4-yl trifluoromethanesulfonate (0.5 g, 1.3 mmol), 5-ethynyl-1,2,3-trimethoxybenzene (0.25 g, 1.3 mmol), Pd(PPh_3_)_4_ (0.1 g, 0.086 mmol) and CuI (0.02 g, 0.11 mmol) under an nitrogen atmosphere. Then, degassed diisopropylamine (i-Pr_2_NH) (30 mL) was added and the mixture heated at 60 °C for 24 h under nitrogen. The resulting dark brown, turbid solution obtained was concentrated to an oil before chromatography on silica using using ethylacetate/hexane (1:4, *v*/*v*) eluent to give the desired product as a white powder. Yield, 0.29 g, 53%. ^1^H NMR (500 MHz; CDCl_3_): δ = 8.67 (d, 2H, *J* = 4.5 Hz, Py-H), 8.59 (d, 2H, *J* = 7.5 Hz, Py-H), 8.52 (s, 2H, Py-H), 7.85 (td, 2H, *J* = 7.5, 1.5 Hz, Py-H), 7.32 (m, 2H, Py-H), 6.76 (s, 2H, Ph-H), 3.84 (s, 6H, −OCH_3_), 3.79 (s, 3H, −OCH_3_).

Ligand **L3**: A mixture of [2,2′:6′,2″]terpyridine-4′-ol (0.5 g, 2.0 mmol), 5-(bromomethyl)-1,2,3-trimethoxybenzene (0.6 g, 2.3 mmol), and K_2_CO_3_ (1.0 g, 7.2 mmol) in dry DMF (50 mL) was heated at 100 °C for 12 h. After cooling to room temperature, the precipitate was filtered out and the solvent was removed under reduced pressure. The residue was subjected to chromatography on silica using ethylacetate/hexane (1:4, *v*/*v*) eluent to give the desired product as a pale yellow powder. Yield, 0.32 g, 37%. ^1^H NMR (300 MHz; CDCl_3_): δ = 8.74 (dt, 2H, *J* = 4.8, 0.87 Hz, Py-H), 8.68 (dt, 2H, *J* = 8.0, 1.0 Hz, Py-H), 8.18 (s, 2H, Py-H), 7.92 (td, 2H, *J* = 7.7, 1.8 Hz, Py-H), 7.40 (dd, 1H, *J* = 4.8, 1.2 Hz, Py-H), 7.37 (dd, 1H, *J* = 4.8, 1.2 Hz, Py-H), 7.06 (s, 2H, Ph-H), 5.36 (s, 2H, −OCH_2_–), 3.95 (s, 6H, −OCH_3_), 3.93 (s, 3H, −OCH_3_).

All metal ion complexes were prepared and crystallised for structure determinations by methods essentially identical to those used for [Co(**L1**)_2_](BF_4_)_2_·CH_3_CN·0.5H_2_O [5]. Full details are given for the case of the Fe(II) complex of **L1** only.

[Fe(**L1**)_2_](ClO_4_)_2_·CH_3_CN: **L1** (0.1 g, 0.25 mmol) was dissolved in hot CH_3_CN (10 mL) and Fe(ClO_4_)_2_·H_2_O (0.035 g, 0.13 mmol) in CH_3_CN (5 mL) slowly added. The mixture was stirred for 2 h at 60 °C, then evaporated to dryness under reduced pressure. The purple residue was washed with cold MeOH and dried in air. Yield: 0.11 g. Vapour diffusion of diethyl ether into an acetonitrile solution of the product gave purple crystals suitable for an X-ray structure determination. ^1^H NMR (300 MHz; CDCl_3_): δ = 9.18 (s, 4H, Py-H), 8.71 (dt, 4H, *J* = 8.0, 0.75 Hz, Py-H), 7.97 (td, 4H, *J* = 7.7, 1.5 Hz, Py-H), 7.58 (s, 4H, Ph-H), 7.23 (dt, 4H, *J* = 5.6, 0.7 Hz, Py-H), 7.14 (dd, 2H, *J* = 5.6, 1.3 Hz, Py-H), 7.12 (dd, 2H, *J* = 5.6, 1.3 Hz, Py-H), 4.15 (s, 12H, −OCH_3_), 3.95 (s, 6H, −OCH_3_). Anal. Calcd. for C_50_H_45_Cl_2_FeN_7_O_14_: C, 54.86; H, 4.14; N, 8.96. Found: C, 54.80; H, 4.16; N, 9.01%.

[Ni(**L1**)_2_](ClO_4_)_2_·CH_3_CN: Substitution of Ni(ClO_4_)_2_.6H_2_O (0.46 g, 0.13 mmol) for Fe(ClO_4_)_2_·H_2_O in the above procedure gave a pale yellow powder. Yield: 0.10 g. Anal. Calcd. for C_50_H_45_Cl_2_NiN_7_O_14_: C, 54.72; H, 4.13; N, 8.93. Found: C, 54.76; H, 4.12; N, 8.90%.

[Co(**L2**)_2_](BF_4_)_2_·H_2_O: Use of **L2** (0.1 g, 0.24 mmol) and Co(BF_4_)_2_.6H_2_O (0.04 g, 0.12 mmol) gave a deep orange powder, Yield: 0.12 g. Anal. Calcd. for C_52_H_44_B_2_CoF_8_N_6_O_7_: C, 56.91; H, 4.04; N, 7.66. Found: C, 56.95; H, 4.01; N, 7.60%.

[Cd(**L2**)_2_](ClO_4_)_2_·CH_3_CN·Et_2_O: Use of **L2** (0.1 g, 0.24 mmol) and Cd(ClO_4_)_2_.H_2_O (0.04 g, 0.12 mmol) gave a pale yellow powder. Yield: 0.09 g. ^1^H NMR (300 MHz; CDCl_3_): δ = 8.82 (s, 4H, Py-H), 8.65 (d, 4H, *J* = 8.1 Hz, Py-H), 8.27 (td, 4H, *J* = 7.8, 1.7 Hz, Py-H), 8.13 (d, *J* = 4.6 Hz, 4H, Py-H), 7.55 (dd, 4H, *J* = 5.1, 0.84 Hz, Py-H), 7.53 (dd, 2H, *J* = 5.1, 0.72 Hz, Py-H), 7.08 (s, 4H, Ph-H), 3.94 (s, 12H, −OCH_3_), 3.83 (s, 6H, −OCH_3_). Anal. Calcd. for C_58_H_55_CdCl_2_N_7_O_15_: C, 54.71; H, 4.35; N, 7.70. Found: C, 54.80; H, 4.26; N, 7.75%.

[Co(**L3**)_2_](BF_4_)_2_: Use of **L3** (0.1 g, 0.24 mmol) and Co(BF_4_)_2_·6H_2_O (0.04g, 0.12 mmol) gave an orange powder. Yield: 0.11 g. Anal. Calcd. for C_52_H_48_B_2_CoF_8_N_4_O_8_: C, 57.32; H, 4.44; N, 5.14. Found: C, 56.98; H, 4.41; N, 5.10%.

[Cu(**L3**)_2_](ClO_4_)_2_·CH_3_CN: Use of **L3** (0.1 g, 0.23 mmol) and Cu(ClO_4_)_2_·6H_2_O (0.043 g, 0.12 mmol) gave a green powder. Yield: 0.12 g. Anal. Calcd. for C_54_H_51_Cl_2_CuN_5_O_16_: C, 55.89; H, 4.43; N, 6.03. Found: C, 56.01; H, 4.39; N, 6.11%.

### 3.3. Crystallography

For all complexes other than [Fe(**L1**)_2_](ClO_4_)_2_·CH_3_CN, crystals were coated with Paratone-*N* oil and the diffraction data measured at temperatures between 100 and 294 K depending on the particular species with synchrotron radiation (λ in the range 0.62988–0.85000 Å depending on the crystal) on an ADSC Quantum-210 detector at 2D SMC with a silicon (111) double crystal monochromator (DCM) at the Pohang Accelerator Laboratory, Korea. The ADSC Q210 ADX program [23] was used for data collection (detector distance 63 mm, omega scan; Δω = 1°, exposure time 1 s per frame) and HKL3000sm (Version 703r) [24] was used for cell refinement, data reduction and absorption correction. For [Fe(**L1**)_2_](ClO_4_)_2_·CH_3_CN, data were collected at 173(2) K using graphite-monochromated MoK_α_ radiation with a CCD area detector on a Bruker APEX2 diffractometer [25]. The structures were solved by direct methods, [26] and refined by full-matrix least-squares refinement on *F^2^* using SHELXL-97 [27]. The positions of all non-hydrogen atoms were refined with anisotropic displacement factors. All hydrogen atoms were placed using a riding model, and their positions were constrained relative to their parent atoms using the appropriate HFIX command in SHELXL-2013. Summary data are given in Table 2; full information can be obtained free of charge from the Cambridge Crystallographic Data Centre *via* www.ccdc.cam.ac.uk/data_request/cif by citing CCDC 955464–955470.

## 4. Conclusions

The use of Hirshfeld surfaces calculated from single-crystal X-ray structure determinations is a convenient means of identifying labile interactions in metal complexes of functionalised terpyridine ligands. It shows that peripheral interactions of aromatic groups in cationic complexes with their counteranions, *viz*., interactions of the aromatic–CH…base type, should generally be considered more important influences upon the lattice structures than those arising from any face-to-face contacts of aromatic groups within separate cations. This is consistent with known theoretical calculations for systems even where there are such contacts giving rise to the terpyridine embrace [8,9]. It is a more difficult but probably more significant issue in relation to properties such as solid state magnetism to establish how a given lattice may distort to accommodate dimensional changes at a metal centre but it is hoped that the extended study of crystallographic information along the lines presently described should provide a worthwhile contribution to its mastery.

## Supplementary Information



## Figures and Tables

**Table 1:** Co–N bond lengths/Å in [Co(**L3**)_2_](BF_4_)_2_.

Bond	100(2) K (low spin)	294(2) K (high spin)
Co–N1	1.983(1)	2.138(3)
Co–N2	1.871(1)	1.996(3)
Co–N3	2.002(1)	2.119(4)
Co–N4	2.163(1)	2.163(4)
Co–N5	1.935(1)	1.997(3)
Co–N6	2.169(1)	2.146(3)

**Table 2:** Crystal and refinement data.

	[Fe(L1)_2_](ClO_4_)_2_·CH_3_CN	[Ni(L1)_2_](ClO_4_)_2_·CH_3_CN	[Co(L2)_2_](BF_4_)_2_·H_2_O	[Cd(L2)_2_](ClO_4_)_2_·CH_3_CN·Et_2_O	[Cu(L3)_2_](ClO_4_)_2_·CH_3_CN	[Co(L3)_2_](BF_4_)_2_	[Co(L3)_2_](BF_4_)_2_
Empirical formula	C_50_H_45_Cl_2_FeN_7_O_14_	C_50_H_45_Cl_2_N_7_NiO_14_	C_52_H_42_B_2_CoF_8_N_6_O_7_	C_58_H_55_CdCl_2_N_7_O_15_	C_54_H_52_Cl_2_CuN_8_O_16_	C_50_H_46_B_2_CoF_8_N_6_O_8_	C_50_H_46_B_2_CoF_8_N_6_O_8_
Formula weight	1094.68	1097.54	1095.47	1273.39	1203.47	1091.48	1091.48
Crystal system	Triclinic	Triclinic	Triclinic	Triclinic	Triclinic	Triclinic	Triclinic
Space group	*P-1*	*P-1*	*P-1*	*P-1*	*P-1*	*P-1*	*P-1*
Color	Violet	Brown	Brown	Green	Blue	Violet	Violet
Crystal size/mm^3^	0.30 × 0.20 × 0.10	0.20 × 0.15 × 0.05	0.60 × 0.20 × 0.20	0.40 × 0.20 ×0.20	0.25 × 0.05 × 0.05	0.15 × 0.10 × 0.04	0.15 × 0.10 × 0.04
*a*/Å	9.5687(1)	9.524(2)	12.1236(7)	11.679(2)	9.468(2)	12.913(3)	13.195(3)
*b*/Å	12.8164(2)	12.744(3)	14.1679(7)	14.919(3)	11.740(2)	13.071(3)	13.245(3)
*c*/Å	20.6974(3)	20.896(4)	18.7880(9)	18.546(4)	25.516(5)	15.749(3)	16.037(3)
α/°	91.228(1)	91.40(3)	69.932(1)	70.86(3)	80.38(3)	102.03(3)	98.96(3)
β/°	101.151(1)	100.88(3)	89.557(2)	80.39(3)	89.84(3)	98.33(3)	101.43(3)
γ/°	107.052(1)	106.62(3)	71.613(2)	74.71(3)	76.99(3)	107.56(3)	108.45(3)
*V*/Å^3^	2372.58(6)	2378.3(9)	2,858.1(3)	2933.3(10)	2722.7(10)	2416.3(8)	2532.8(9)
*Z*	2	2	2	2	2	2	2
ρ_calc_/g cm^−3^	1.532	1.533	1.273	1.442	1.468	1.500	1.431
λ/Å	0.71073	0.62988	0.71073	0.6300	0.8000	0.65000	0.65000
*λ*/K	173(2)	102(2)	153(2)	102(2)	102(2)	100(2)	294(2)
μ/mm^−1^	0.509	0.432	0.378	0.389	0.796	0.448	0.428
*F*(000)	1132	1136	1122	1308	1246	1122	1122
θ_min-max_	1.67–28.35	1.765–33.388	3.00–27.47	1.83–29.49	0.46–29.59	1.55–33.28	1.22–27.09
Reflections collected	41,675	39,046	28,444	40,426	18,452	30,929	14,656
Independent reflections (*R*_int_)	11,681 (0.0481)	19,720 (0.0215)	13,012 (0.0594)	20,465 (0.0102)	9463 (0.0367)	16.065 (0.0227)	7942 (0.0336)
Reflections with *I* > 2σ(*I*)	8029	16,887	7570	19,871	7544	13,193	5658
Goodness-of-fit on *F*^2^	1.041	1.074	1.086	1.026	1.053	1.108	1.030
Final R indices [*I* > 2σ(*I)*]^a^	*R*_1_ = 0.0475	0.0433	*R*_1_ = 0.0744	0.0443	0.0536	*R*_1_ = 0.0414	0.0710
	*wR*_2_ = 0.1131	0.1258	*wR*_2_ = 0.1977	0.1257	0.1493	*wR*_2_ = 0.1208	0.2077
Final R indices [all data] a	*R*_1_ = 0.0802	0.0505	*R*_1_ = 0.1227	0.0451	0.0660	*R*_1_ = 0.0524	0.0942
	*wR*_2_ = 0.1279	0.1304	*wR*_2_ = 0.2379	0.1581	*wR*_2_ = 0.1259	0.2346	

a *R*_1_ = ∑||*F*_o_| − |*F*_c_||/∑|*F*_o_|, *wR*_2_ = [∑*w*(*F*_o_^2^ − *F*_c_^2^)^2^/∑*w*(*F*_o_^2^)^2^]^1/2^.
