# Supplementary material for: Supramolecular Interactions of Terpyridine-Derived Cores of Metallomesogen Precursors
Source: Int J Mol Sci. 2013 Oct 15;14(10):20729–43. doi: 10.3390/ijms141020729 (PMC3821640; doi:10.3390/ijms141020729)
Supplement: Supplementary file 1 [file ijms-14-20729-s001.pdf]

Supplementary Information

Figure S1. Magnetic susceptibility measurements for [Co(**L2**)$_2$](BF$_4$)$_2$•H$_2$O.

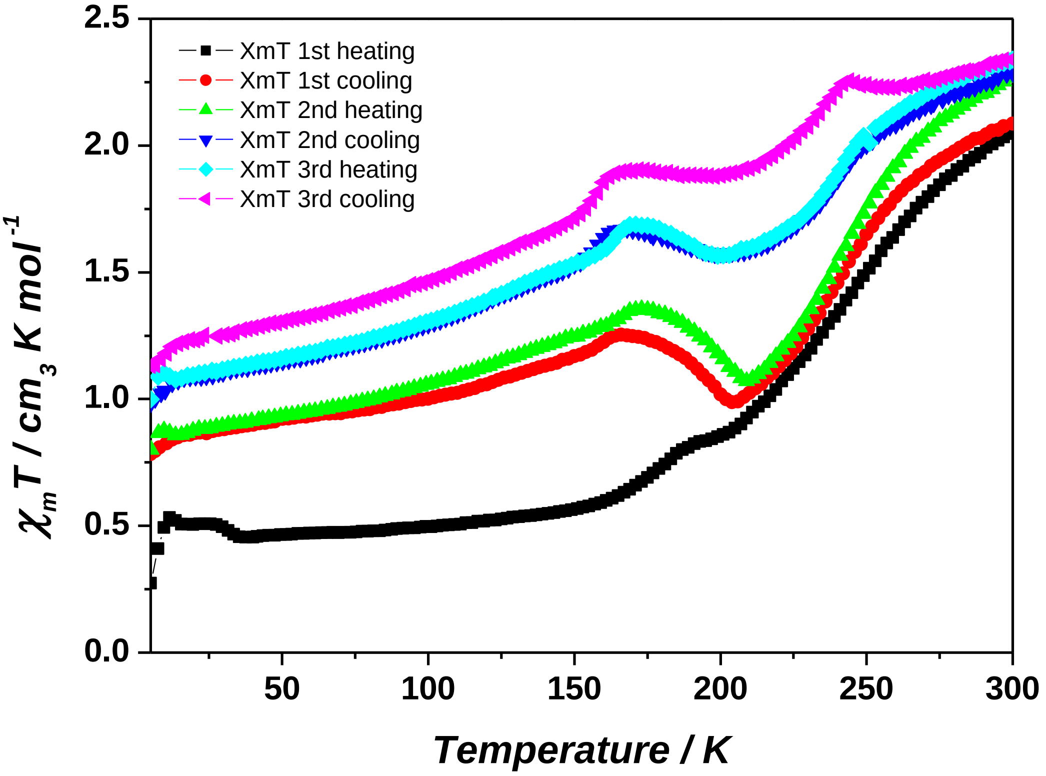

Figure S2. A partial view, down b, of the lattice of [Cu(**L3**)$_2$](ClO$_4$)$_2$•CH$_3$CN.

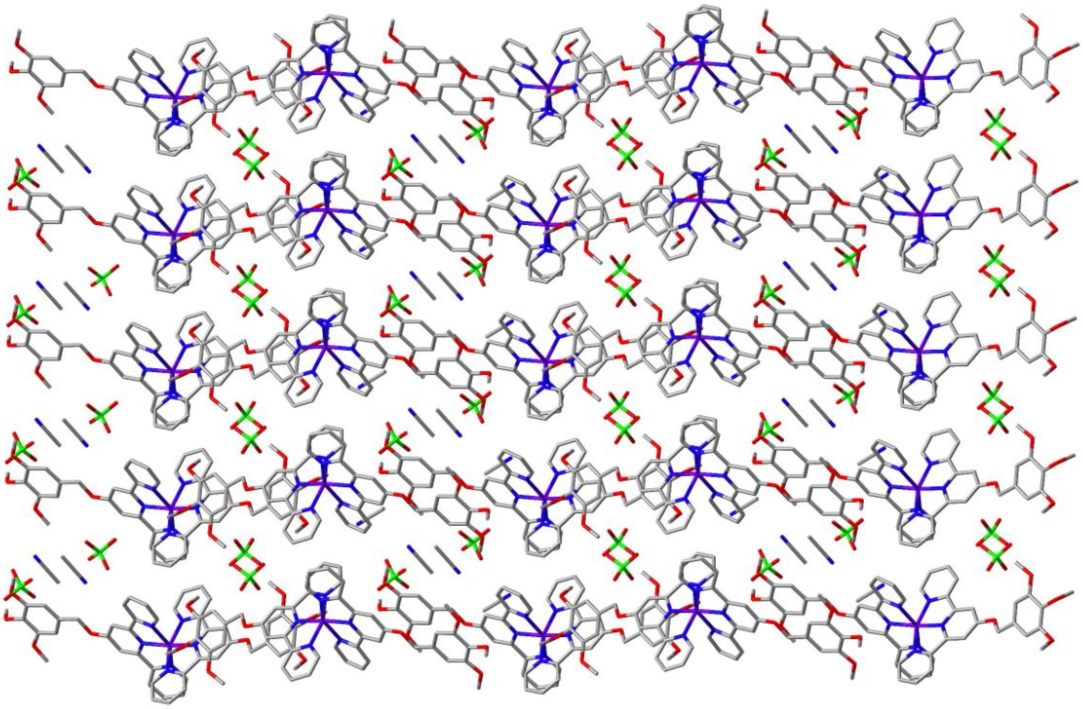

Figure S3. Views, perpendicular to the mean plane of the more nearly planar ligand, of the various cations studied in the present work.

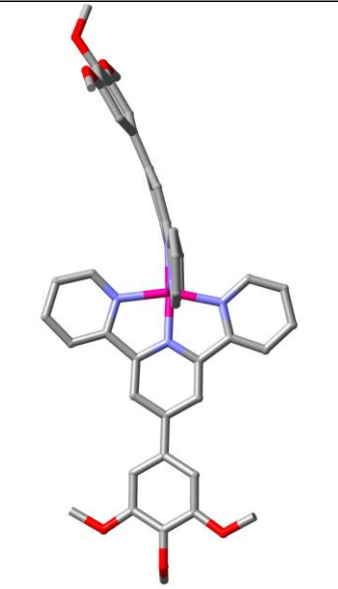	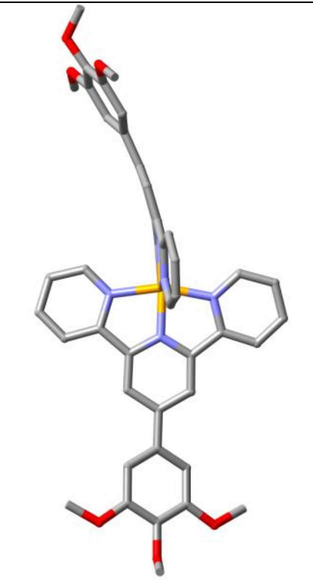	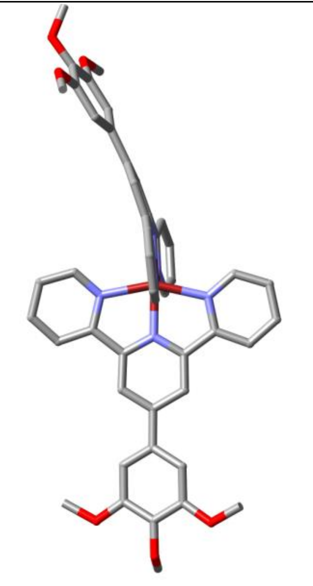	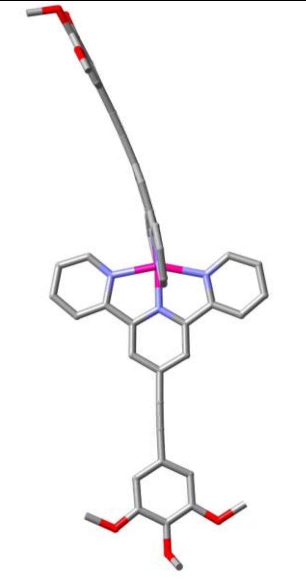
$[Co(\mathbf{L1})_2](BF_4)_2$•$CH_3CN$•$0.5H_2O$	$[Fe(\mathbf{L1})_2](ClO_4)_2$•$CH_3CN$	$[Ni(\mathbf{L1})_2](ClO_4)_2$•$CH_3CN$	$[Co(\mathbf{L2})_2](BF_4)_2$•$H_2O$

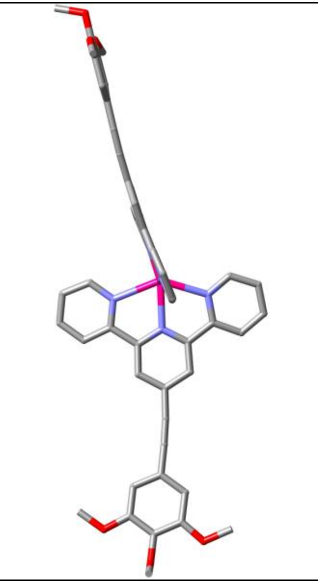	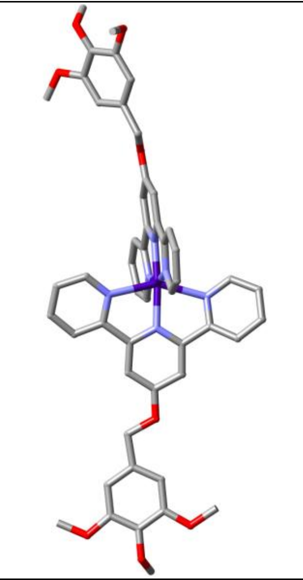	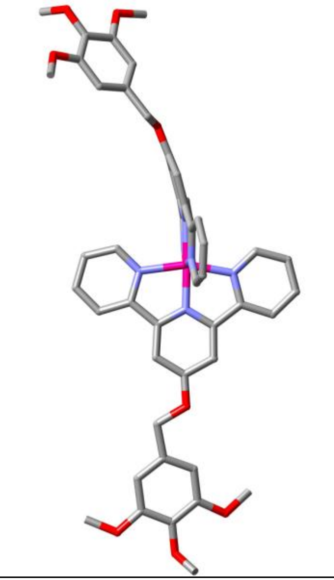	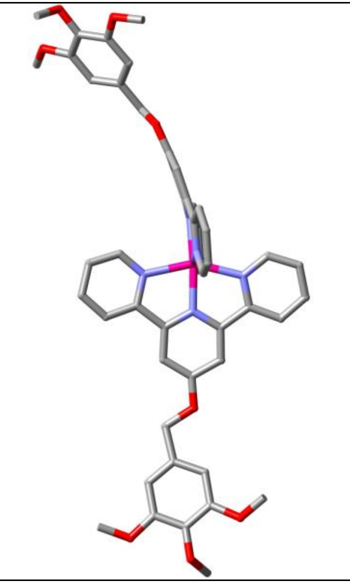
$[Cd(\mathbf{L2})_2](BF_4)_2$•$CH_3CN$•$Et_2O$	$[Cu(\mathbf{L3})_2](ClO_4)_2$•$CH_3CN$	$[Co(\mathbf{L3})_2](BF_4)_2$, 100 K	$[Co(\mathbf{L3})_2](BF_4)_2$, 294 K

© 2013 by the authors; licensee MDPI, Basel, Switzerland. This article is an open access article distributed under the terms and conditions of the Creative Commons Attribution license (http://creativecommons.org/licenses/by/3.0/).
